# Bioremediation of acetamiprid and sulfoxaflor co-contamination by *Ensifer* sp. DA6 and characterization of a novel nitrile hydratase involved

**DOI:** 10.3389/fmicb.2025.1705774

**Published:** 2025-11-11

**Authors:** Wenlong Yang, Jia Kang, Yahui Shao, Yun Geng, Yingxin Zhang, Renlu Liu, Gao Chen

**Affiliations:** 1 State Key Laboratory of Nutrient Use and Management, Shandong Academy of Agricultural Sciences, Jinan, China; 2 Engineering Research Center of Jinan for Agricultural Microbial Resource Conservation and Biomanufacturing, Institute of Crop Germplasm Resources, Shandong Academy of Agricultural Sciences, Jinan, China; 3 Key Laboratory of Jiangxi Province for Functional Biology and Pollution Control in Red Soil Regions, School of Life Sciences, Jinggangshan University, Ji'an, China.

**Keywords:** biodegradation, nitrile hydratase, insecticide, acetamiprid, sulfoxaflor

## Abstract

**Introduction:**

The co-contamination of acetamiprid (ACE) and sulfoxaflor (SUL) threatens ecosystem security, yet their microbial remediation remains unexplored.

**Methods:**

The bacterium Ensifer sp. DA6, which transforms ACE to IM-1-2 and SUL to X11719474, was isolated. Its genome was analyzed, and the nitrile hydratase (NHase) gene cluster was heterologously expressed in E. coli. The NHase was purified and modeled.

**Results:**

Immobilized Ensifer sp. DA6 degraded ACE and SUL in Yellow River water. The recombinant E. coli and purified NHase both acquired ACE/SUL degradation ability. The NHase is a cobalt-containing enzyme with a and β subunits plus an accessory protein, and its active site was predicted.

**Discussion:**

This is the first report on microbial co-degradation of ACE and SUL, identifying a novel NHase as the key enzyme, providing a potential bioremediation strategy.

## Graphical Abstract

**Figure:**
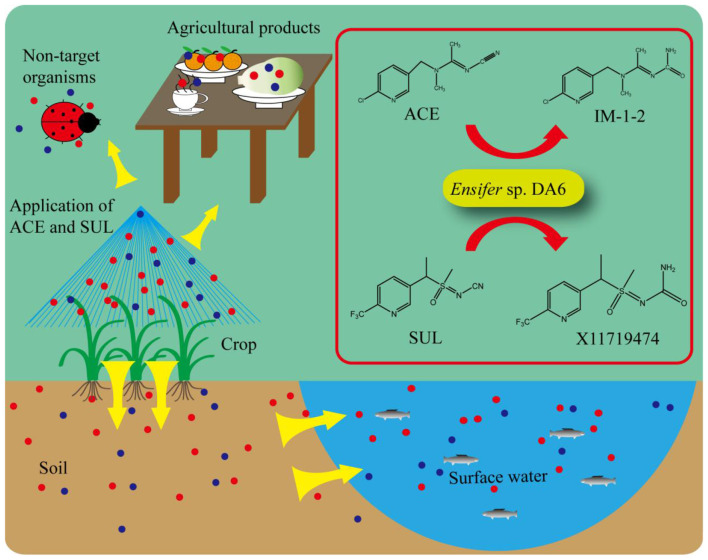

## 1 Introduction

Acetamiprid {ACE, *N*-[(6-chloro-3-pyridyl)methyl]-*N*′-cyano-*N*-methylacetamidine}, a neonicotinoid insecticide, acts as a selective agonist targeting insect nicotinic acetylcholine receptors (nAChRs). This compound selectively binds insect-specific nAChR subunits, mimicking acetylcholine binding to cause persistent receptor activation and disrupt neurotransmission (Tomizawa et al., 2000). ACE is used for the control of pests such as aphids, rice planthoppers, and leafhoppers in crops including cotton, wheat, rice, and apples (Saha et al., 2017; Saggioro et al., 2019; Zhang et al., 2022). Sulfoxaflor {SUL, X14422208, [*N*-(methyloxido{1-[6-(trifluoromethyl)-3-pyridinyl] ethyl}-λ^4^-sulfanylidene) cyanamide]} is a novel sulfoximine insecticide. Similar to neonicotinoids, SUL targets insect nicotinic acetylcholine receptors (nAChRs), enabling effective control of sap-feeding insect pests (Watson et al., 2011). SUL is applied against pests including *Aphis gossypii* Glover, *Philaenus spumarius*, and *Laodelphax striatellus* on crops such as brown rice and lettuce (Chen et al., 2016; Kim et al., 2016; Xu et al., 2016; Chung et al., 2017; Dader et al., 2019).

However, ACE exhibits high desorption (reaching 96.3%), suggesting poor soil retention that enhances bioavailability and elevates translocation risks to environmental compartments (Lalín-Pousa et al., 2025). ACE exposure induces significant developmental toxicity in zebrafish embryos, causing mortality and spinal deformities (e.g., bent spine), thereby highlighting its ecological risk to aquatic vertebrates (Ma et al., 2019). ACE poses long-term risks to honey bee health even at sublethal concentrations (Shi et al., 2025). Chronic ACE exposure induces persistent toxicological effects in *Oreochromis mossambicus*, suggesting human risks through the consumption of ACE-exposed fish (Raj and Joseph, 2015). ACE induces clinical symptoms in mice, such as decreased body weight, respiratory depression, and hepatic effects (Chakroun et al., 2016). SUL exhibits toxicity to non-target species including *Amblyseius swirskii*, bumblebees, and earthworms (Fang et al., 2018; Siviter et al., 2018; Dáder et al., 2019). Furthermore, SUL residues in rats and mice, animal models for human diseases, raise significant concerns due to their toxicity. High-dose SUL exposure during gestation in rats induced neonatal survival reduction and fetal abnormalities, primarily limb contractures (Ellis-Hutchings et al., 2014). High-dose dietary SUL caused hepatotoxicity in mice (Lebaron et al., 2014). Therefore, environmental ACE and SUL residues present significant health risks.

Photocatalytic degradation (using TiO_2_ and light produced by a Xe lamp) and oxidation during Fenton's reactions have been used to remove ACE from water samples (Mitsika et al., 2013; Borges et al., 2016). Physical and chemical degradation methods require extreme conditions, are expensive, and cause environmental contamination. SUL was either not degraded or only slightly degraded during hydrolysis and was not photodegraded in acidic water or sterilized soil (Yang et al., 2022). Regarding agricultural production inputs, approximately 82% of pesticides undergo biodegradation, with microorganisms playing a pivotal role in pesticide degradation (Maggi et al., 2023). Microbial remediation serves as a critical environmental remediation method for organic pollutants, demonstrating substantial application prospects and potential (Eisenstein, 2025). Compared to chemical and physical methods, microbial remediation exhibits economic efficiency, operational simplicity, and minimal environmental impact, aligning with ecological civilization and sustainable development strategies. Many microorganisms have been found to degrade ACE, such as the bacterium *Actinomycetes Streptomyces canus* CGMCC 13662, *Stenotrophomonas maltophilia* CGMCC 1.1788, and *Variovorax boronicumulans* CGMCC4969, as well as the yeast *Rhodotorula mucilaginosa* (Dai et al., 2010; Guo et al., 2019). Many microbes have been found to degrade SUL, such as *Aminobacter* sp. CGMCC 1.17253 and *Pseudomonas stutzeri* CGMCC 22915 (Yang et al., 2020; Jiang et al., 2022). However, no studies have investigated the degradation of ACE and SUL co-contaminants to date.

This approach leverages natural microbial metabolic activities to transform pollutants into less toxic or non-toxic substances through enzymatic degradation pathways. ACE, a traditionally widely used insecticide with a high market share, leaves significant environmental residues, while SUL, a novel insecticide, is also showing increasing environmental persistence, leading to heightened risks of co-contamination. This study aimed to screen microorganisms that could remediate ACE and SUL co-contamination and subsequently characterize the metabolic pathways and enzymes involved in ACE and SUL degradation. The findings of this study will enhance the understanding of ACE and SUL degradation by microbes in the environment and may aid the development of a novel bacteria-based method for ACE and SUL co-contamination bioremediation.

## 2 Materials and methods

### 2.1 Chemicals and media

ACE (98% purity) and SUL (95% purity) were obtained from Hubei Zhengxingyuan Fine Chemical Co., *Ltd*. (Wuhan, China). IM-1-2 was prepared using the method described by Zhou et al. (2014). X11719474 was prepared following the method of Yang et al. (2020). Analytical-grade reagents were procured from Sinopharm Chemical Reagent Co., *Ltd*. (Shanghai, China). High-performance liquid chromatography (HPLC)-grade acetonitrile was purchased from Tedia Company, *Inc*. (Fairfield, OH, USA). The lysogeny broth (LB) medium was formulated with 5 g yeast extract, 10 g NaCl, and 10 g tryptone dissolved in 1 L of deionized water (pH adjusted to 7.2). The mineral salt medium (MSM), specifically formulated for screening ACE- and SUL-degrading microorganisms, was prepared according to Liu et al. (2011). For the solid culture medium, LB agar plates were prepared by supplementing the LB formulation with 2% (w/v) bacteriological agar.

### 2.2 Enrichment of ACE- and SUL-degrading microbial consortia from saline-alkaline soil

Soil samples were collected from saline-alkaline lands in Binzhou, Shandong Province, China. For microbial enrichment, 1.0 g of soil was suspended in 20 mL of sterile MSM broth in a 100 mL Erlenmeyer flask containing five sterile glass beads (5 mm diameter). The soil solution was homogenized by orbital shaking at 200 rpm for 1 h at 30 °C. Primary enrichment was initiated by transferring 1 mL of the soil suspension to 80 mL of sterile MSM broth supplemented with 200 mg/L each of ACE and SUL in a 250 mL Erlenmeyer flask. The culture was maintained under aerobic conditions with continuous shaking (220 rpm) at 30 °C for 14 d. Secondary enrichment was subsequently performed by inoculating 1 mL of the primary culture into fresh MSM broth containing elevated concentrations of the target compounds (500 mg/L each of ACE and SUL) in a new 250 mL flask. This secondary culture was incubated under identical conditions (220 rpm, 30 °C) for an additional 14 d.

### 2.3 Isolation and identification of ACE- and SUL-degrading bacterial and genomic DNA sequencing

The enriched bacterial fluid was then spread on LB agar plates after being diluted 10,000- and 100,000-fold, and the plates were incubated at 30 °C. Morphologically distinct single colonies were selected, streaked onto LB agar plates, and subsequently incubated at 30 °C to obtain pure cultures. The degradation capacities of the isolated strains for ACE and SUL were evaluated using resting cell assays. A bacterial isolate was inoculated into 20 mL of LB medium contained in a 100 mL flask. The culture was incubated at 30 °C (220 rpm) for 24 h. After an incubation period of 24 h, a 2 mL aliquot of the culture was transferred to a 500 mL flask containing 100 mL of LB medium. The flask was then incubated at 30 °C and 220 rpm for 24 h. Cell pellets were collected by centrifugation at 7,000 × *g* for 5 min. The cell pellets were washed twice with sterilized phosphate-buffered saline (PBS; 50 mmol/L, pH 7.0). Subsequently, they were resuspended in PBS (50 mmol/L, pH 7.0) containing 200 mg/L ACE and 200 mg/L SUL, respectively. The cell suspension density at optical density at 600 nm (OD_600_) was adjusted to 5. The cell suspensions were aliquoted into pre-sterilized 50 mL centrifuge tubes (2 mL per tube). Following sealing with breathable film, the tubes were incubated at 30 °C with 220 rpm agitation for the indicated duration. The solution was centrifuged at 10,000 × *g* for 5 min to pellet the cells, and 700 μL of the clarified supernatant was transferred to a clean microtube. An aliquot of 300 μL of acetonitrile was added to the supernatant and thoroughly mixed. The resulting mixture was filtered through a 0.22 μm membrane prior to HPLC analysis. Morphological characterization and 16S rRNA gene sequencing were employed as complementary approaches to identify ACE- and SUL-degrading bacterial isolates. Light microscopic observation of Gram-stained cells was performed to determine cellular morphology. Selected clones were subjected to colony polymerase chain reaction (PCR) for targeted amplification of the 16S rRNA gene. The amplification primers were 27F (5′-AGAGTTTGATCCTGGCTCAG-3′) and 1492R (5′-TACGGTTACCTTGTTACGACTT-3′). The amplicons were then sequenced by Sangon Biotech Co., *Ltd*. (Shanghai, China). The 16S rRNA gene sequences of ACE- and SUL-degrading isolates were deposited in the GenBank database. The nucleotide sequence homology of the strain DA6 was analyzed using the Basic Local Alignment Search Tool for nucleotides (BLASTn) against the GenBank non-redundant database. Phylogenetic reconstruction was conducted in MEGA 6 by constructing a neighbor-joining tree with 1,000 bootstrap replicates, based on aligned 16S rRNA gene sequences of closely related type strains. Whole-genome sequencing and annotation were conducted by OneMore Technology Co., *Ltd*. (Wuhan, China).

### 2.4 High-performance liquid chromatography (HPLC) analysis

ACE, SUL, and their metabolites were analyzed using a Waters 600E HPLC system equipped with an HC-C18 column (4.6 mm × 250 mm, 5 μm; Agilent, USA). HPLC was achieved at a flow rate of 1.0 mL/min using a mobile phase comprising water containing 0.01% (v/v) acetic acid and acetonitrile (70:30, v/v). Detection wavelengths were set at 235 nm and 220 nm.

### 2.5 Liquid chromatography-mass spectrometry (LC-MS) analysis

The metabolic products of ACE and SUL were identified through the LC-MS analysis of the DA6-transformed samples. This was performed using an Agilent 1290 Infinity LC system coupled with a 6460 Triple Quadrupole MS (Agilent Technologies, Santa Clara, CA), equipped with an electrospray ion source operating in both positive and negative ion modes. HPLC analysis was performed under the conditions described above, except with a flow rate of 0.6 mL/min.

### 2.6 Degradation kinetics of ACE and SUL mediated by resting *Ensifer* sp. DA6 cells

*Ensifer* sp. DA6 was cultured as previously described, except for supplementing 0.1 mmol/L CoCl_2_. Subsequently, resting cells were used to individually transform ACE and SUL and to co-transform both substrates under identical conditions. Samples were collected daily for four consecutive days, and HPLC analysis followed the aforementioned method.

### 2.7 Immobilized *Ensifer* sp. DA6 degraded ACE and SUL in surface water

Surface water was sampled from the Yellow River in Jinan, China. The samples were filtered through a 0.22 μm membrane and amended with ACE and SUL to achieve a final concentration of 100 mg/L each, preparing transformation solutions. Immobilized cells were prepared by inoculating 3 mL of seed culture into 300 mL of LB medium supplemented with 0.1 mmol/L CoCl_2_ in a 1,000 mL Erlenmeyer flask. After incubation at 30 °C with shaking (220 rpm) for 20 h, the culture was centrifuged at 7,000 rpm for 5 min (4 °C). The harvested cells were washed twice with PBS (50 mmol/L, pH 7.0) and resuspended in 10 mL sterile water. The resuspended cells were combined with 30 mL of a 4% (w/v) sodium alginate solution, stirred for 10–20 min until homogeneous, and adjusted to a final alginate concentration of 3%. The alginate solution was dispensed into a 2% (w/v) CaCl_2_ solution using a 1 mL syringe, forming gel beads approximately 3 mm in diameter. The beads were then incubated in the CaCl_2_ solution at 4 °C for 24 h to complete gelation. After washing the beads three times with sterile water to remove residual Ca^2^^+^ ions, the immobilized cells reached a density of 3.6 × 10^9^ cells per gram. Subsequently, 14 g of these beads were transferred into a 500 mL flask containing 100 mg/L ACE and SUL, with parallel controls established: (1) bacterial control (surface water + alginate beads with cells) and (2) substrate control (surface water + substrates + alginate beads). The flasks were sealed with breathable sealing film and maintained at 30 °C with agitation at 220 rpm. Every 24 h, samples were collected and centrifuged at 13,200 rpm for 10 min, and 700 μL of the supernatant was mixed with 300 μL of acetonitrile. After sterile filtration through a 0.22 μm membrane, the samples were subjected to HPLC analysis.

### 2.8 Cloning and over-expression of nitrile hydratase (NHase) genes in *E. coli* Rosetta (DE3)

Total Genomic DNA was extracted from the ACE- and SUL-degrading isolate using a MiniBEST Bacterial Genomic DNA Extraction Kit (TaKaRa Bio Inc., Dalian, China). The primers used for the amplification of NHase genes, which contained *Eco*RI and *Xho*I restriction enzyme sites, were Primer-F (ACAGCAAATGGGTCGCGGATCCGAATTCATGTCCGAACACCATCATGGCC) and Primer-R (ATCTCAGTGGTGGTGGTGGTGGTGCTCGAGTCAGGGGCGCTCAGGATCG). The primers were synthesized by Sangon Biotech Co., *Ltd*. (Shanghai, China). Reaction mixtures for PCR analysis contained 1 × PrimeSTAR Max Premix (TaKaRa Bio Inc., Dalian, China), forward and reverse primers (1 mmol/L), 1 ng of DNA template, and ultrapure water to a final volume of 20 μL. Amplification was conducted using a PCR thermal cycler (TaKaRa Bio Inc., Osaka, Japan) with the following cycling conditions: initial hot start at 95 °C for 1 min, followed by 32 cycles of denaturation at 98 °C for 10 s, annealing at 56 °C for 10 s, and extension at 72 °C for 30 s, concluding with a final extension phase at 72 °C for 10 min. The amplified DNA fragments were subjected to electrophoretic analysis on 1% (w/v) agarose gels prepared in 1 × TAE buffer, and the gels were stained with StarGreen safe Nucleic Acid Dye (GenStar) to visualize DNA bands. After successful verification, the *Eco*RI/*Xho*I-digested PCR products were ligated into the expression vector pET28a using the ClonExpress MultiS One Step Cloning Kit (Vazyme Biotech Co., *Ltd*., Nanjing, China) according to the manufacturer's instructions. The resultant recombinant plasmid carrying the NHase gene was individually transformed into competent *E. coli* Rosetta cells, following the transformation protocol described by Sun et al. (2016).

### 2.9 Degradation of ACE and SUL co-contaminants by resting *E. coli* Rosetta (DE3) cells overexpressing NHase

The expression of the NHase gene in recombinant *E. coli* Rosetta (DE3) was induced by the simultaneous addition of 0.2 mmol/L isopropyl β-D-thiogalactoside (IPTG) and 0.2 mmol/L cobalt chloride (CoCl_2_). After incubation at 37 °C for 6 h, the enzymatic activity of NHase-overexpressing *E. coli* Rosetta (DE3) cells against ACE and SUL was determined using a resting cell biodegradation assay (as described previously). The samples were then incubated at 30 °C with shaking at 200 rpm for 10 min. The culture supernatants were subsequently collected by centrifugation at 12,000 × *g* for 10 min to remove cellular debris, after which the clarified supernatants were subjected to HPLC analysis, as described previously.

### 2.10 Enzyme purification and biochemical characterization

The expression of the NHase gene in recombinant *E. coli* Rosetta (DE3) was induced as described above. After incubation at 37 °C for 6 h, the N-terminal 6 × His-tagged NHase protein overexpressed in *E. coli* Rosetta (DE3) was purified using Ni-NTA Agarose affinity chromatography according to the manufacturer's protocol (Sangon Biotech Co. *Ltd.*, Shanghai, China). The protein samples were denatured by heating at 100 °C for 5 min in sodium dodecyl sulfate (SDS) sample buffer containing SDS, β-mercaptoethanol, and bromophenol blue. Subsequently, sodium dodecyl sulfate polyacrylamide gel electrophoresis (SDS-PAGE) was performed using a 12% separating gel and a 5% stacking gel, with an initial voltage of 80 V, followed by 120 V upon sample entry into the separating gel. The gel was fixed in 40% methanol/10% acetic acid for 30 min and stained with 0.1% Coomassie Brilliant Blue R-250 in 50% methanol/10% acetic acid for 1 h. The gel was sequentially processed by washing with deionized water to remove excess unbound dye, followed by immersion in a destaining buffer containing 50% methanol and 10% acetic acid, and incubated at room temperature on a shaker. One unit (U) of enzyme activity was defined as the amount of enzyme required to catalyze the generation of 1 μmol of IM-1-2 or X11719474 in 1 min. The optimal pH for SUL degradation by the NHase was determined using a standardized reaction system containing 10 μL of the purified NHase (4.78 mg/mL) and 990 μL of the SUL solution (100 mg/L) in 50 mmol/L buffer (PBS, CA/SC, or Tis-HCl) at 37 °C with constant agitation at 200 rpm for 20 min. Gradient pH conditions (5.0–9.0) were established by adjusting buffer compositions: CA/SC (pH 5.0–6.0), PBS (pH 6.0–8.0), and Tis-HCl (pH 7.0–9.0). The optimal temperature for SUL degradation by the NHase was determined by incubating reaction mixtures containing 10 μL of the purified NHase (4.78 mg/mL) and 990 μL of the SUL solution in PBS (50 mmol/L, pH 7.0) at 37 °C with constant agitation at 200 rpm for 20 min. The kinetic parameters of the NHase for ACE degradation were determined by analyzing reaction mixtures (1 mL total volume) containing 10 μL of the purified NHase (4.78 mg/mL) and 990 μL of the ACE solution (25–1,000 mg/L) in PBS (50 mmol/L, pH 7.0) at 45 °C, with continuous orbital shaking at 200 rpm for 60 min. The kinetic parameters of NHase for SUL degradation were determined by incubating a 1 mL reaction mixture containing 8 μL of the purified NHase (4.78 mg/mL) and 992 μL of the SUL solution (25–1,000 mg/L) in PBS (50 mmol/L, pH 7.0) at 45 °C, with constant orbital shaking at 200 rpm for 30 min. After the reaction was completed, 500 μL of the reaction solution was collected, followed by the addition of 300 μL of acetonitrile to quench the reaction and 200 μL of PBS (50 mmol/L, pH 7.0). All reactions were analyzed using HPLC, as previously described. The kinetic parameters for the NHase-catalyzed degradation of ACE and SUL were determined through non-linear regression analysis using the OriginPro 9 software with the Michaelis–Menten enzyme kinetics module.

### 2.11 Three-dimensional homology modeling of the *Ensifer* sp. DA6 NHase

A three-dimensional homology model of the *Ensifer* sp. DA6 NHase was constructed using the SWISS-MODEL platform (https://swissmodel.expasy.org/interactive). The co-type nitrile hydratase crystal structure (PDB: 3QZ5, resolution 2.5 Å) from *Pseudomonas putida* was used as the template for constructing the NHase model. For the alpha and beta subunits, the sequence identity was 62.49% and 43.58%, while the similarity was 0.49 and 0.41, respectively. The quality of the constructed NHase structure was assessed using GMQE and QMEANDisCo (Waterhouse et al., 2018). The modeled NHase structure was analyzed using the molecular visualization software Chimera (https://www.cgl.ucsf.edu/chimera/). Active sites were predicted through sequence alignment with reference nitrile hydratase sequences.

### 2.12 Molecular docking of the *Ensifer* sp. DA6 NHase with ACE and SUL

Molecular docking was performed using the CB-Dock2 platform (https://cadd.labshare.cn/cb-dock2/index.php), a blind docking tool that integrates cavity detection, structure-based docking with AutoDock Vina, and template-based docking using homologous templates to enhance prediction accuracy. The binding sites and affinities between the *Ensifer* sp. DA6 NHase and the ligands ACE and SUL were evaluated to elucidate potential interaction mechanisms. Subsequently, molecular dynamics simulations were conducted to assess the stability and conformational behavior of the docked complexes under physiological conditions. This comprehensive computational approach provides insight into the molecular interactions that may underlie the enzyme–ligand recognition process.

### 2.13 Molecular dynamics simulation

Molecular dynamics simulations were conducted using the online server iMODS (https://imods.iqf.csic.es/) (López-Blanco et al., 2014). This tool employs an internal coordinate system to analyze collective modes of motion. The iMODS platform demonstrates broad compatibility across modern web browsers and devices. Upon submission of the PDB file for the docked complex as input, with all parameters maintained at their default configurations, the server rapidly generated results within minutes. Output data encompassed multiple key parameters, including B-factor values, eigenvalue analysis, variance, protein deformability profiles, elastic network representations, and covariance maps.

## 3 Results and discussion

### 3.1 Isolation and identification of ACE- and SUL-degrading microbes and genome sequencing

**Figure 1:**
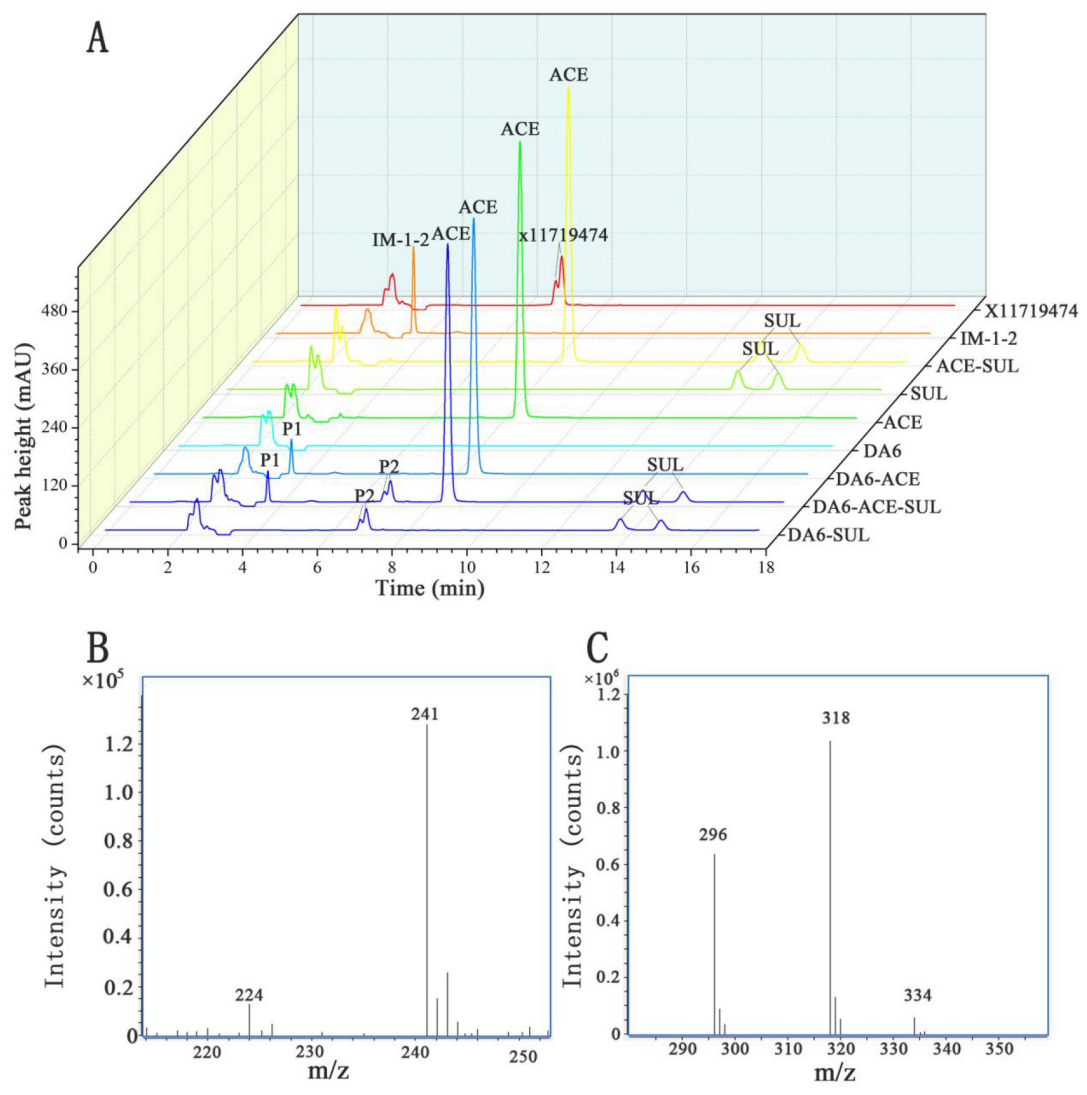
High-performance liquid chromatography (HPLC) and liquid chromatography-mass spectrometry (LC-MS) analysis of the metabolites produced during the degradation of ACE and SUL by the isolate DA6. **(A)** HPLC analysis of the transformation of ACE and SUL by the isolate DA6. **(B)** Positive-ion mode mass spectrometry analysis of the degradation product P1. **(C)** Positive-ion mode mass spectrometry analysis of the degradation product P2.

**Figure 2:**
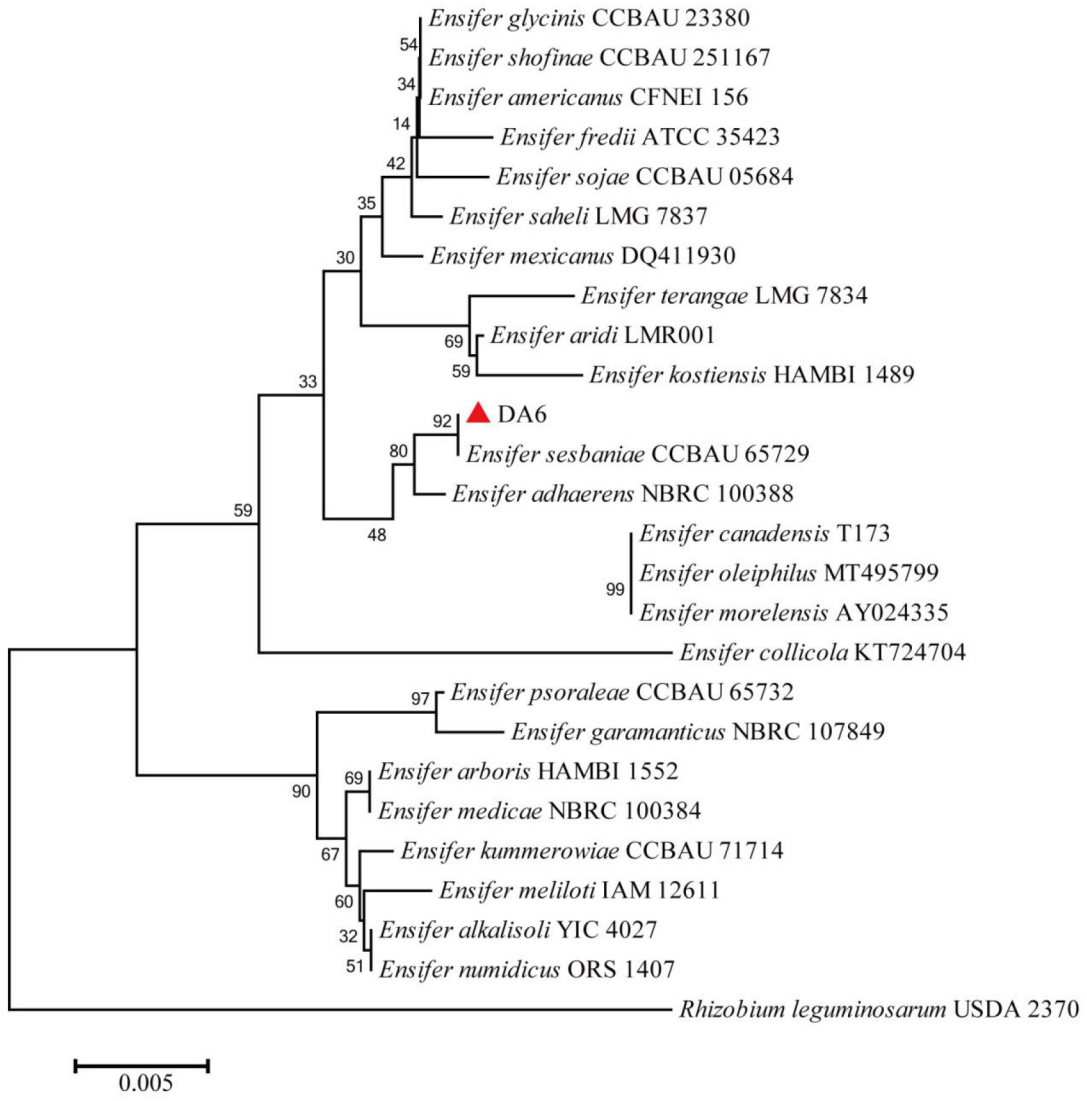
Phylogenetic tree of the isolate DA6. The phylogenetic tree was constructed using the neighbor-joining method, including other members of the genus *Ensifer* and representatives of other taxa, based on 16S rRNA gene sequence comparisons. Bootstrap percentages from 1,000 replicates are shown at the nodes. The sequence of *Rhizobium leguminosarum* USDA 2370 was used as the outgroup.

After dilution and spreading on LB agar plates, 21 morphologically distinct colonies were selected and tested for their ability to degrade ACE and SUL. One isolate, designated DA6, exhibited the ability to degrade both ACE and SUL. An assay for ACE and SUL degradation confirmed that DA6 metabolized ACE and SUL into products labeled P1 and P2, respectively (Figure 1A). The peak heights of SUL and X11719474 were relatively low, while that of ACE was high. When plotting the HPLC chromatograms together using a uniform y-axis scale, the peaks for SUL and X11719474 appeared less sharp and somewhat blunt. The HPLC chromatogram of *Ensifer* sp. DA6-degraded SUL, shown in the Supplementary material, displayed sharp peaks (Supplementary Figure S1). On LB plates, DA6 formed circular, maroon, convex colonies approximately 0.6 mm in diameter with a glistening surface. Gram staining identified the isolate as Gram-negative, with rod-shaped, non-spore-forming colonies observed using optical microscopy. BLAST analysis of the 16S rRNA gene sequence from the isolate DA6 (GenBank accession: SUB15223998) showed high similarity to *Ensifer sesbaniae* CCBAU 65729. A neighbor-joining phylogenetic tree based on the 16S rRNA gene sequences revealed that DA6 clustered within the genus *Ensifer* (Figure 2). DA6 was designated as *Ensifer* sp. DA6 and was stored in the China Center for Type Culture Collection, China General (CCTCC) (Wuhan, China) under the preservation number CCTCC NO: M 20241656. Analysis of the complete genome sequence of *Ensifer* sp. DA6 revealed that there was a nitrile hydratase-encoding gene cluster in the genome (Supplementary Figure S2). The alpha subunit contains a conserved sequence of Val-Cys-Thr-Leu-Cys-Ser-Cys, indicating that the NHase of *Ensifer* sp. DA6 is cobalt-ion-dependent. Microorganisms screened from the environment can effectively degrade many pesticide residues, including clothianidin and flonicamid (Zhang et al., 2023, 2024). A novel axenic bacterium with unique ACE and SUL co-contaminant bioremediation traits was isolated from soil. This finding enhances our understanding of the microbial degradation of co-contaminants ACE and SUL and provides a basis for developing bioremediation agents targeting sulfoxaflor residues in the environment.

### 3.2 Identification of the metabolites

The metabolites of ACE include IM-2-1 {N-[(6-chloropyridin-3-yl)methyl]acetamide)}, IM-1-3 {N-[(6-chloropyridin-3-yl)methyl]-N-methylacetamide}, IM-1-2 [(E)-1-(1-{[(6-chloropyridin-3-yl)methyl](methyl)amino}ethylidene)urea], and IM-1-4 [(6-chloropyrid in-3-yl)-N-methylmethanamine] (Guo et al., 2019). The metabolites of SUL include X11596066 (5-ethyl-2-trifluoromethylpyridine), X11721061 {1-[6-(trifluoromethyl)pyridin-3-yl]ethanol}, X11719474 [*N*-(methyl(oxido){1-[6-(trifluoromethyl)pyridin-3-yl] ethyl}-λ4-sulfanylidene)urea], X11519540 {[5-(1-methylsulfonyl)ethyl]-2-(trifluoromethyl)pyridine}, X11579457 ({5-[1-(S-methylsulfonimidoyl)ethyl]}-2-(trifluoromethyl)pyridine), and 5-ethyl-2-(trifluoromethyl)pyridine (Chung et al., 2017; Cutler et al., 2013; Wiezorek et al., 2019). HPLC analysis revealed that the retention times of the products P1 and P2 (3.7 min and 6.9 min, respectively) matched those of the reference compounds IM-1-2 and X11719474. LC-MS analysis revealed that the metabolite P1 exhibited peaks at 241 and 224 m/z, corresponding to [M+H]^+^ and [M-NH_2_]^+^, respectively (Figure 1B). Therefore, the relative molecular weight of P1 was calculated as 240. The relative molecular weight of IM-1-2 is 240, which is consistent with the calculated relative molecular weight of P1. Metabolite P2 showed peaks at 296, 318, and 334 m/z, corresponding to [M+H]^+^, [M+Na]^+^, and [M+K]^+^, respectively (Figure 1C). Therefore, the relative molecular weight of P2 was calculated as 295. The relative molecular weight of X11719474 is 295, which is consistent with the calculated molecular weight of P2. The amino free radicals of IM-1-2 cause the adjacent bonds to break, generating [M-NH_2_]^+^ and ·NH_2_ free radicals. The compound forms adduct ions with sodium (Na^+^) or potassium (K^+^), which is common in electrospray ionization, resulting in peaks such as [M+Na]^+^ and [M+K]^+^. In the HPLC spectrum, SUL exhibited two peaks and P2 showed a split peak. This is because both SUL and X11719474 are chiral molecules with four optical isomers, leading to peak overlap. However, due to the inherent limitations of this method, we were unfortunately unable to fully resolve the four optical isomers of SUL and P2. In the study by Jiang et al. (2022), the C18 reversed-phase column was similarly unable to separate the four optical isomers of SUL and its metabolites. Complete separation would require a chiral column and corresponding method development, which we have not yet undertaken. Nevertheless, the results still demonstrate *Ensifer* sp. DA6′s degradation effect on SUL. *Ensifer* sp. DA6 converts ACE and SUL into their amide derivatives through the hydrolytic pathway, as evidenced by these results.

### 3.3 Degradation kinetics of ACE and SUL mediated by Resting *Ensifer* sp. DA6 cells

**Figure 3:**
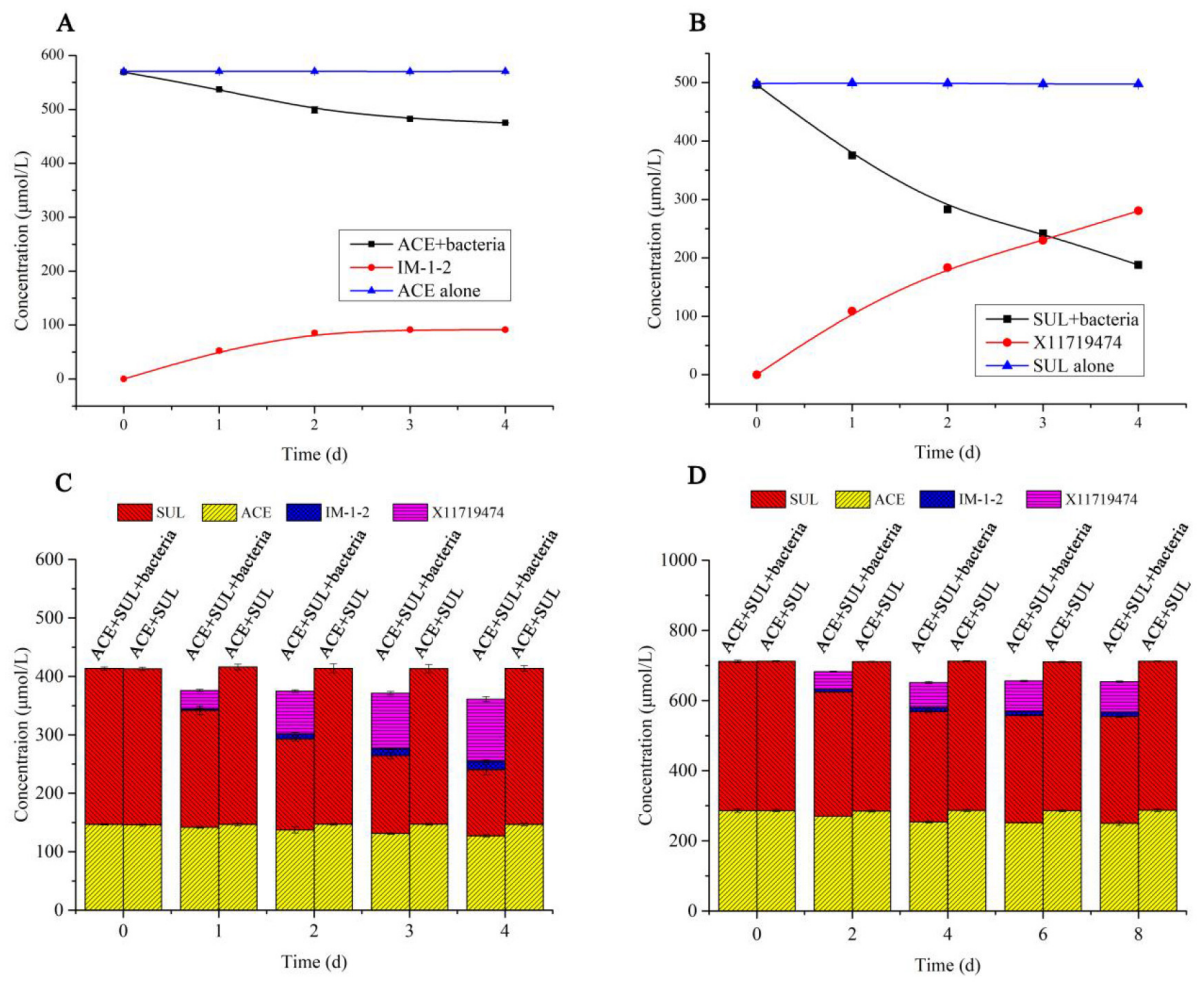
ACE and SUL degraded by *Ensifer* sp. DA6. **(A)** Time course of ACE degradation by resting *Ensifer* sp. DA6 cells. **(B)** Time course of SUL degradation by resting *Ensifer* sp. DA6 cells. **(C)** ACE and SUL degradation by resting *Ensifer* sp. DA6 cells. **(D)** ACE and SUL degradation by immobilized *Ensifer* sp. DA6 cells in surface water.

The degradation rates of ACE and SUL by *Ensifer* sp. DA6 were 5.32% and 14.21%, respectively, over 4 d in the absence of cobalt ions (Table 1). When cobalt ions were added and there was only ACE in the conversion solution, resting *Ensifer* sp. DA6 cells degraded ACE from an initial concentration of 569.09–474.94 μmol/L over 4 d, with a degradation rate of 16.54%, and the degradation half-life was 15.18 d (Figure 3A). When cobalt ions were added and there was only SUL in the conversion solution, resting *Ensifer* sp. DA6 cells degraded SUL from an initial concentration of 496.10–187.85 μmol/L over 4d, with a degradation rate of 62.13%, and the degradation half-life was 2.84 d (Figure 3B). When cobalt ions were added and ACE and SUL were present simultaneously, the degradation rates were 13.44% for ACE and 57.48% for SUL over 4d, and the degradation half-life rates were 18.96 d and 4.00 d, respectively (Figure 3C). When cobalt ions were added, the degradation rate of ACE by *Ensifer* sp. DA6 increased by 2.53-fold compared to the non-cobalt-ion condition, while the degradation efficiency of SUL improved by 4.37-fold. When both ACE and SUL coexisted, the degradation efficiency of ACE by *Ensifer* sp. DA6 decreased by 3.26% compared to the condition with ACE alone, while the degradation efficiency of SUL showed a 4.65% reduction compared to its sole substrate scenario. The coexistence of ACE and SUL exhibited substrate cross-inhibition effects. These results indicated that *Ensifer* sp. DA6 efficiently degraded ACE and SUL into IM-1-2 and X11719474 via the hydration pathway. Although the degradation activity of *Ensifer* sp. DA6 is not very high, it reveals a biodegradation pathway for ACE and SUL residues in the environment.

**Table 1:** Degradation of ACE and SUL by *Ensifer* sp. DA6 under co-free conditions.

**Treatments**	**Concentration (** ***μ*** **mol/L)**			
	**Reduced ACE**	**IM-1-2**	**Reduced SUL**	**X11719474**
DA6-ACE-SUL	6.73 ± 2.07	11.77 ± 0.42	43.80 ± 2.27	41.31 ± 0.89
ACE-SUL	–	–	–	–

### 3.4 Immobilized *Ensifer* sp. DA6 degraded ACE and SUL in surface water

When ACE and SUL were present simultaneously, immobilized *Ensifer* sp. DA6 degraded 5.49% of ACE and 28.33% of SUL in Yellow River surface water over 8 d. The degradation half-lives of ACE and SUL were 98.18 d and 16.65 d, respectively (Figure 3D). Although the immobilized *Ensifer* sp. DA6 cells exhibited relatively low degradation efficiency during ACE and SUL co-contamination remediation, this microbial system nevertheless provides a viable approach for the bioremediation of co-existing pollutants. When *Ensifer* sp. DA6 is actually applied to degrade the co-contaminants ACE and SUL in surface water, various factors such as the pH value, salt content, organic matter content, and environmental temperature of the water may have an impact on the degradation effect. Further research is still needed to understand these influences. This study provides a critical reference value for the ecological protection of surface water systems.

### 3.5 Resting *E. coli* Rosetta (DE3) cells overexpressing NHase degrade ACE and SUL co-contaminants

**Figure 4:**
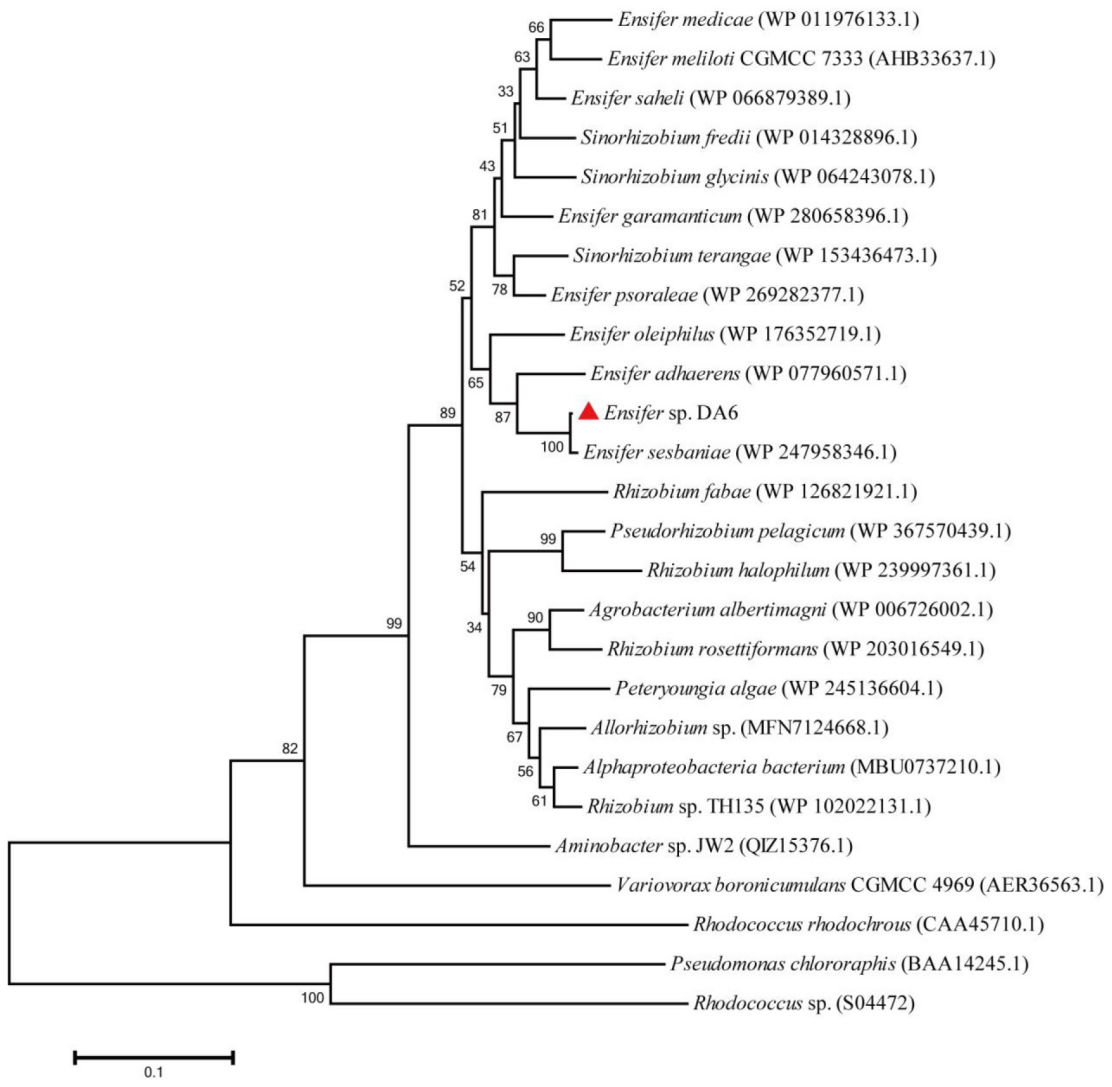
Phylogenetic tree of the NHase α-subunit from *Ensifer* sp. DA6. Bootstrap percentages from 1,000 replicates are shown at the nodes. The NHases from *Pseudomonas chlororaphis* and *Rhodococcus* sp. are iron-type NHases.

**Figure 5:**
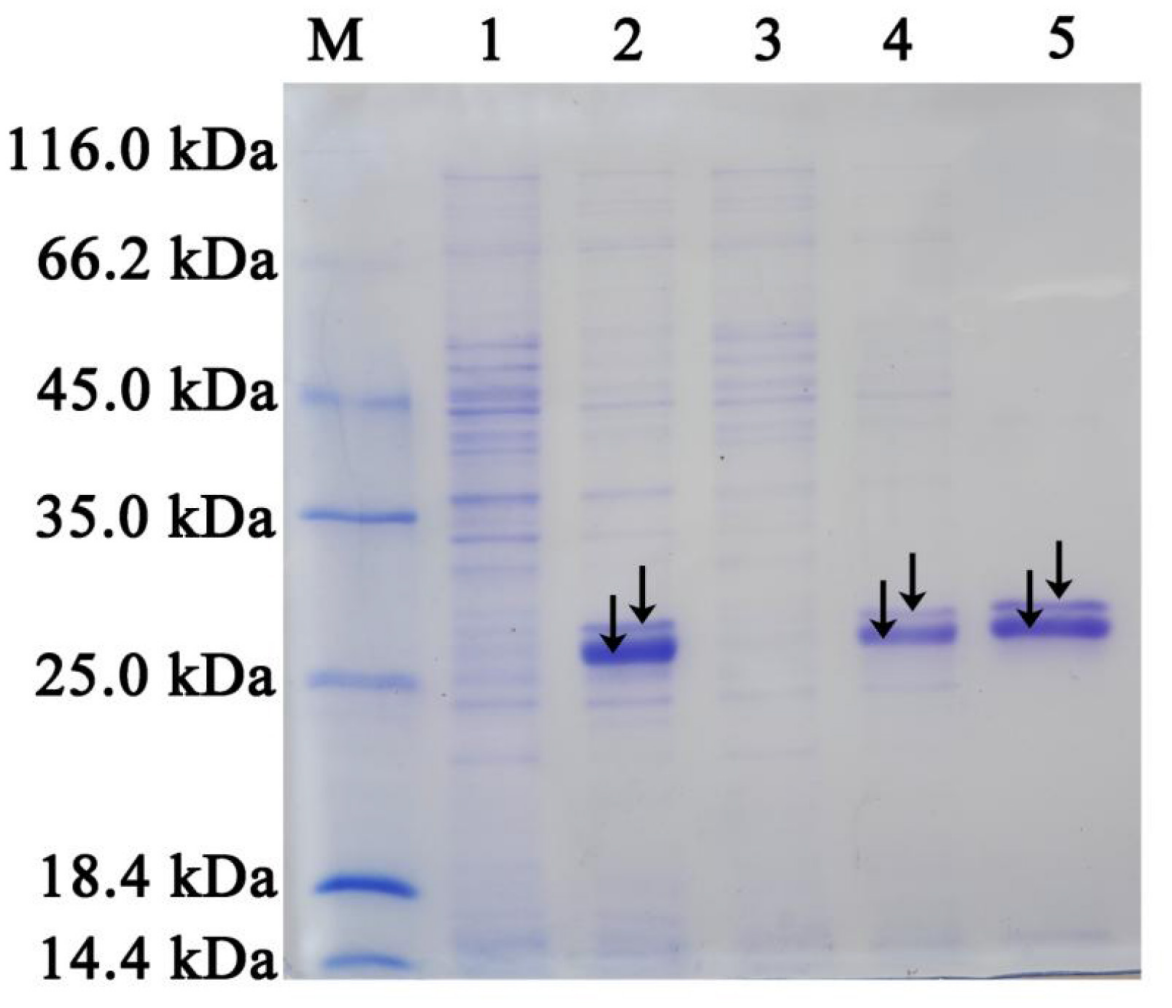
SDS-PAGE analysis of the NHase overexpressed in *E. coli* Rosetta (DE3), along with the purified NHase. Lanes 1 and 3: Total protein extracts from *E. coli* Rosetta (DE3) strains containing pET28a (control) and pET28a-*NHase*, respectively. Lanes 2 and 4: Soluble protein fractions from *E. coli* Rosetta (DE3) strains containing pET28a (control) and pET28a-*NHase*, respectively. Lane 5: Purified NHase with an N-terminal His-tag. Lane M: Standard protein markers (116.0, 66.2, 45.0, 35.0, 25.0, 18.4, and 14.4 kDa). The alpha (the smaller band marked) and beta (the larger band marked) subunits were clearly separated by SDS-PAGE. However, the accessory protein was not detected.

IM-1-2 and X11719474 are metabolites of ACE and SUL generated via the hydration pathway and require NHase activity. The NHase gene from *Ensifer* sp. DA6 (GenBank: α-subunit MN381727, β-subunit MN381728, accessory protein MN381729) was amplified and ligated into pET28a (Supplementary Figures S3, S4). Then, the plasmid was transformed into *E. coli* Rosetta (DE3), and this engineered strain was labeled as D1. The phylogenetic tree confirmed clustering of the *Ensifer* sp. DA6 NHase with *E. sesbaniae* CCBAU 65729 (Figure 4). Protein expression was induced and confirmed by SDS-PAGE (Figures 5 and Supplementary Figure S5). D1 degraded 16.85% of ACE and 68.35% of SUL individually within 10 min. In coexisting conditions, it degraded 12.58% of ACE and 40.07% of SUL within 10 min (Table 2). D1 exhibited enzymatic behavior consistent with *Ensifer* sp. DA6, confirming NHase-mediated hydration of ACE and SUL to IM-1-2 and X11719474. Similar to NHase-mediated degradation of ACE/SUL by *Ensifer* sp. DA6, other cyano-containing pesticides (e.g., thiacloprid) can be transformed into amides via NHase-mediated hydration. This is the first study on NHase-catalyzed co-contaminant degradation of ACE and SUL by microorganisms.

**Table 2:** Degradation of ACE and SUL by D1.

**Treatments**	**Concentration (** ***μ*** **mol/L)**			
	**Reduced ACE**	**IM-1-2**	**Reduced SUL**	**X11719474**
D1-ACE	54.53 ± 2.10	48.68 ± 0.43	–	–
*E. Coli* Rosetta (DE3)-ACE	–	–	–	–
ACE	–	–	–	–
D1-SUL	–	–	199.37 ± 12.23	176.37 ± 4.84
*E. Coli* Rosetta (DE3)-SUL	–	–	–	–
SUL	–	–	–	–
D1-ACE-SUL	40.98 ± 3.89	38.69 ± 0.78	177.32 ± 6.50	170.90 ± 3.16
*E. Coli* Rosetta (DE3)*-*ACE	–	–	–	–
ACE-SUL	–	–	–	–

### 3.6 Enzymatic characterization of the *Ensifer* sp. DA6 NHase

**Figure 6:**
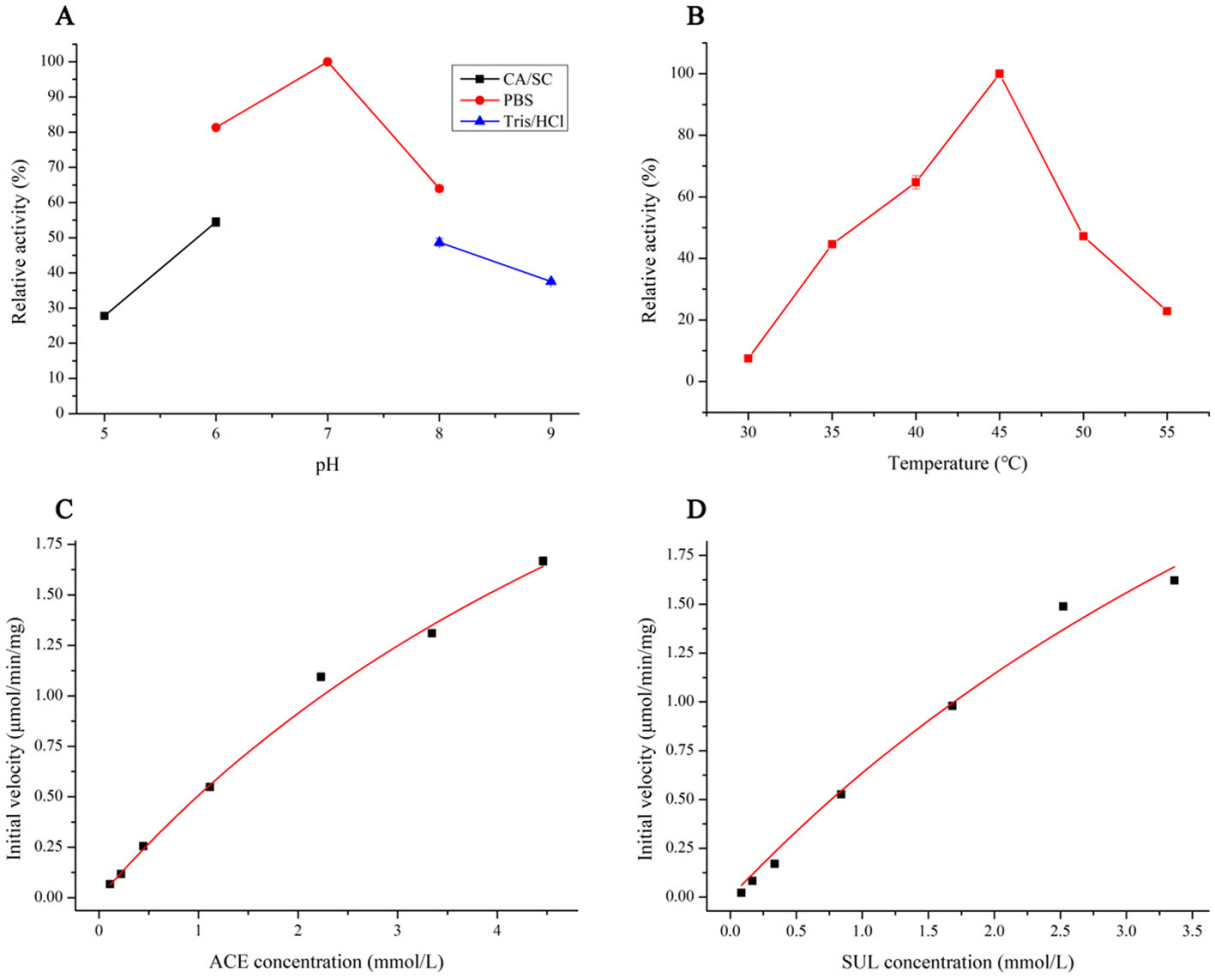
Enzymatic characterization and kinetic parameters of ACE and SUL degradation reactions catalyzed by the NHase. **(A)** Effects of pH on NHase activity. **(B)** Effects of temperature on NHase activity. **(C)** Kinetic parameters for the hydration of ACE degraded by the NHase. **(D)** Kinetic parameters for the hydration of SUL degraded by NHase.

The *Ensifer* sp. DA6 NHase was expressed and purified (Figure 5). The optimal pH for SUL degradation by the NHase was 7.0, defined as 100% activity (Figure 6A). Activity toward SUL decreased significantly below pH 7.0 or above pH 7.0; therefore, this NHase exhibits higher activity at neutral pH. The optimal temperature for SUL degradation by the NHase was 45 °C, defined as 100% activity (Figure 6B). Activity toward SUL decreased significantly below 45 °C or above 45 °C. Kinetic parameter analysis indicated that the degradation of ACE and SUL by the NHase exhibited classic Michaelis–Menten kinetics. For the NHase-catalyzed degradation of ACE, *V*_*max*_ was 4.64 mU/mg and *Km* was 8.16 mmol/L (Figure 6C); for SUL, *V*_*max*_ was 5.68 mU/mg and *Km* was 7.93 mmol/L (Figure 6D).

### 3.7 Homology modeling of the *Ensifer* sp. DA6 NHase

**Figure 7:**
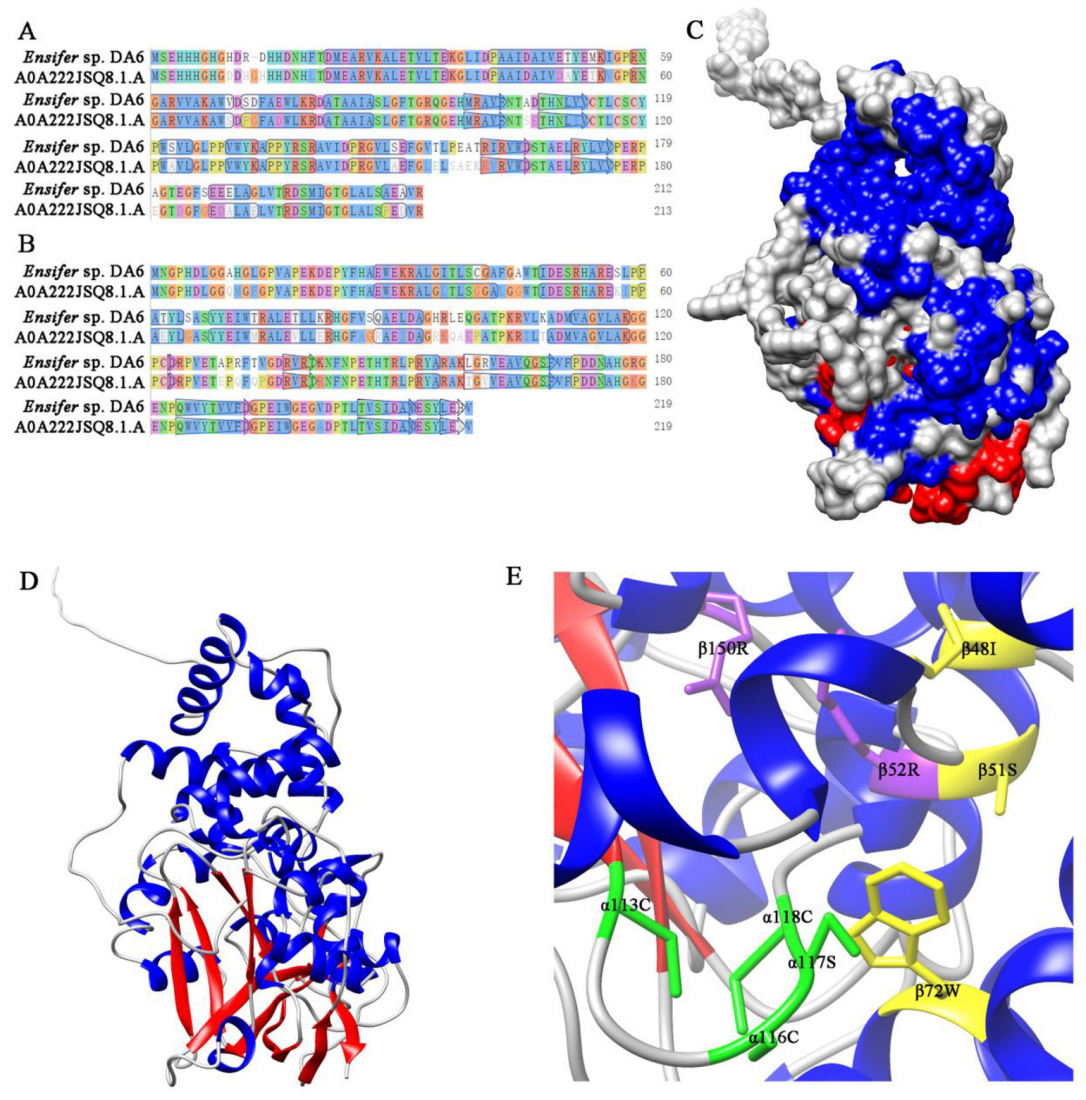
Alignment of the NHase with the template sequence and the three-dimensional homology model of the NHase. **(A)** Alignment of the NHase α-subunit with the template sequence. **(B)** Alignment of the NHase β-subunit with the template sequence. **(C)** Three-dimensional homology model of the NHase (surface representation). **(D)** Three-dimensional homology model of the NHase (strand representation). **(E)** Active sites of the NHase. Amino acid residues marked in green constitute the putative cobalt ion-binding sites. Amino acid residues marked in yellow participate in the recognition of the substrate and form a hydrophobic pocket. Amino acid residues marked in purple form hydrogen bonds stabilizing the claw setting.

The three-dimensional homology model of NHase was built using SWISS-MODEL, based on the *P. putida* NHase crystal structure (Figures 7A–D). The GMQE scores for α- and β-subunits were 0.86 and 0.79, respectively; QMEANDisCo scores were 0.83 ± 0.06 and 0.73 ± 0.06, respectively. Sequence identity between the NHase and the model was 62.93% for the α-subunit and 83.56% for the β-subunit. Based on sequence alignments with selected NHases (Supplementary Figures S6, S7), αCys-113, αCys-116, αSer-117, and αCys-118 coordinate cobalt binding; βArg52 and βArg150 stabilize the claw setting via hydrogen bonds; βIle-48, βSer-51, and βTrp-72 form a hydrophobic pocket for substrate recognition (Figure 7E).

### 3.8 Molecular docking and molecular dynamics simulation of the *Ensifer* sp. DA6 NHase with ACE and SUL

**Figure 8:**
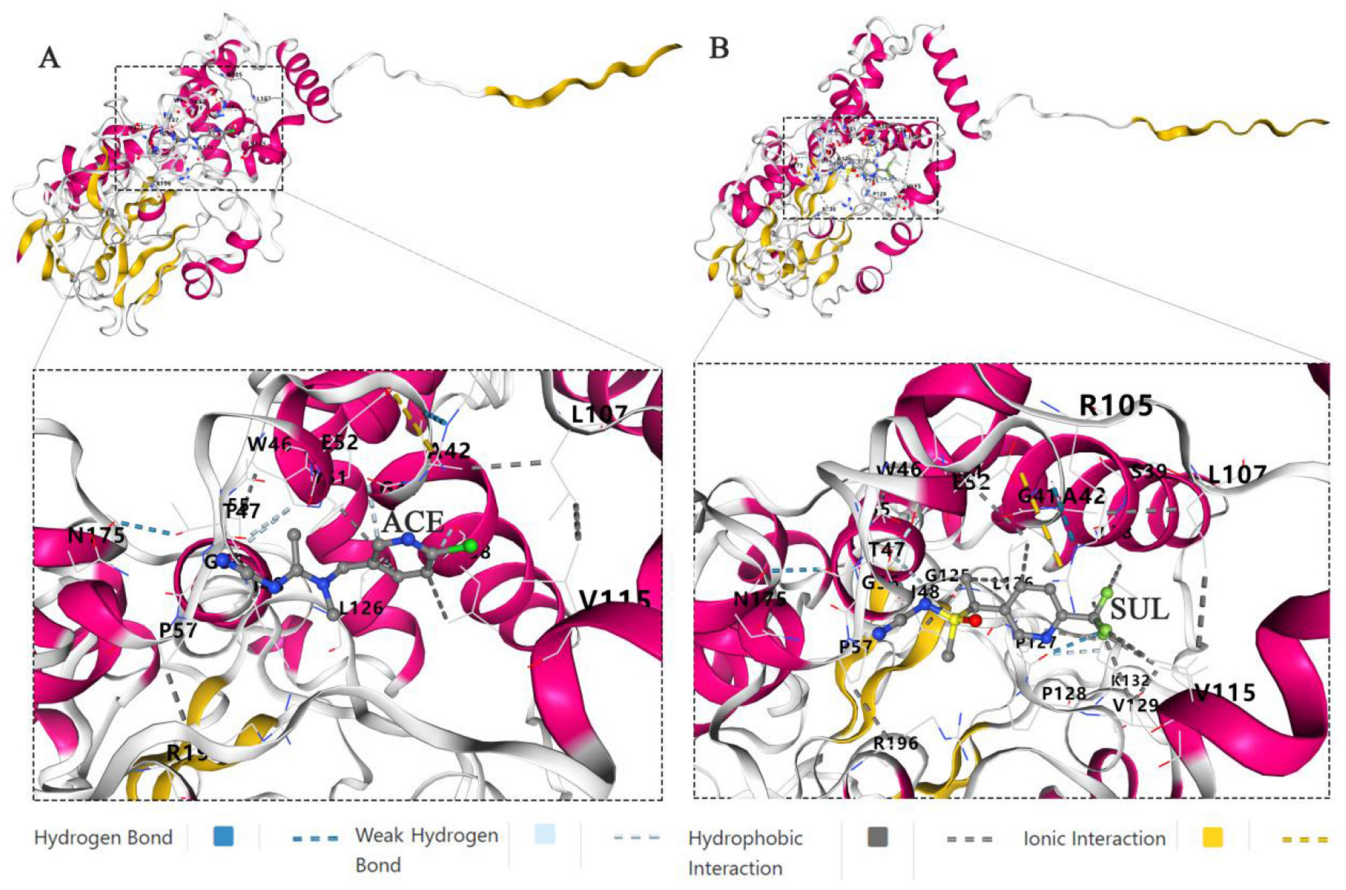
Docking and molecular dynamics simulation. **(A)** Docking and molecular dynamics simulation of the *Ensifer* sp. DA6 NHase and ACE. **(B)** Docking and molecular dynamics simulation of the *Ensifer* sp. DA6 NHase and SUL.

Molecular docking and molecular dynamics simulations of the *Ensifer* sp. DA6 NHase with the ligands ACE and SUL were successfully conducted using the CB-Dock2 platform. Molecular docking revealed Vina scores of −7.6 kcal/mol for ACE and −8.0 kcal/mol for SUL bound to the *Ensifer* sp. DA6 NHase. Lower (more negative) Vina scores correlate with enhanced binding stability. Scores ≤ -5.0 kcal/mol are generally indicative of potential binding activity, confirming that the *Ensifer* sp. DA6 NHase has a high binding affinity for both ACE and SUL. Molecular interaction analysis revealed that ACE formed hydrogen bonds with αGly56, βAsn175, and βIle48; weak hydrogen bonds with αTyr, βLeu38, and βGly41; hydrophobic interactions with αTyr, αLeu126, and βLeu38; and an ionic interaction between αGlu and βArg105 (Figure 8A). Molecular interaction analysis revealed that SUL formed hydrogen bonds with αLeu126, βLeu38, βIle48, βVal115, and βLeu116; an ionic interaction between αGlu52 and βArg105; additional hydrogen bonds between αPro127 and αPro128, as well as between βAla42 and βArg105; and weak hydrogen bonds between αTyr51 and βIle48, αIle55 and αGly125, and αPro127 and αPro128 (Figure 8B).

### 3.9 Molecular dynamics simulation

**Figure 9:**
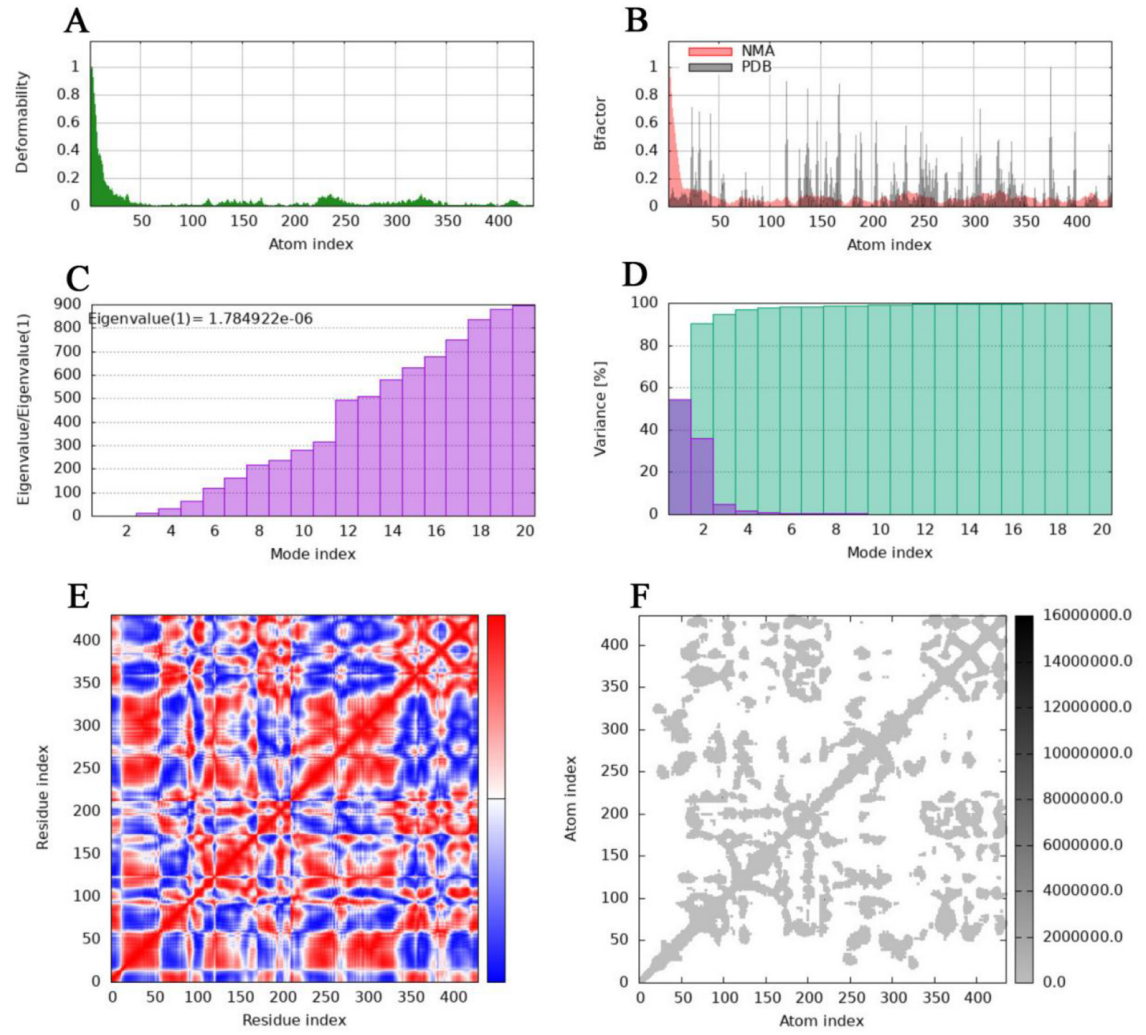
Molecular dynamics simulation of the NHase-ACE complex. **(A)** Main-chain deformability. **(B)** Experimental B-factor. **(C)** Eigenvalues. **(D)** Variance: green indicates cumulative variance, and purple indicates individual variance. **(E)** Covariance map: correlated motion is shown in red, and anticorrelated motion is shown in blue, respectively. **(F)** Elastic network.

**Figure 10:**
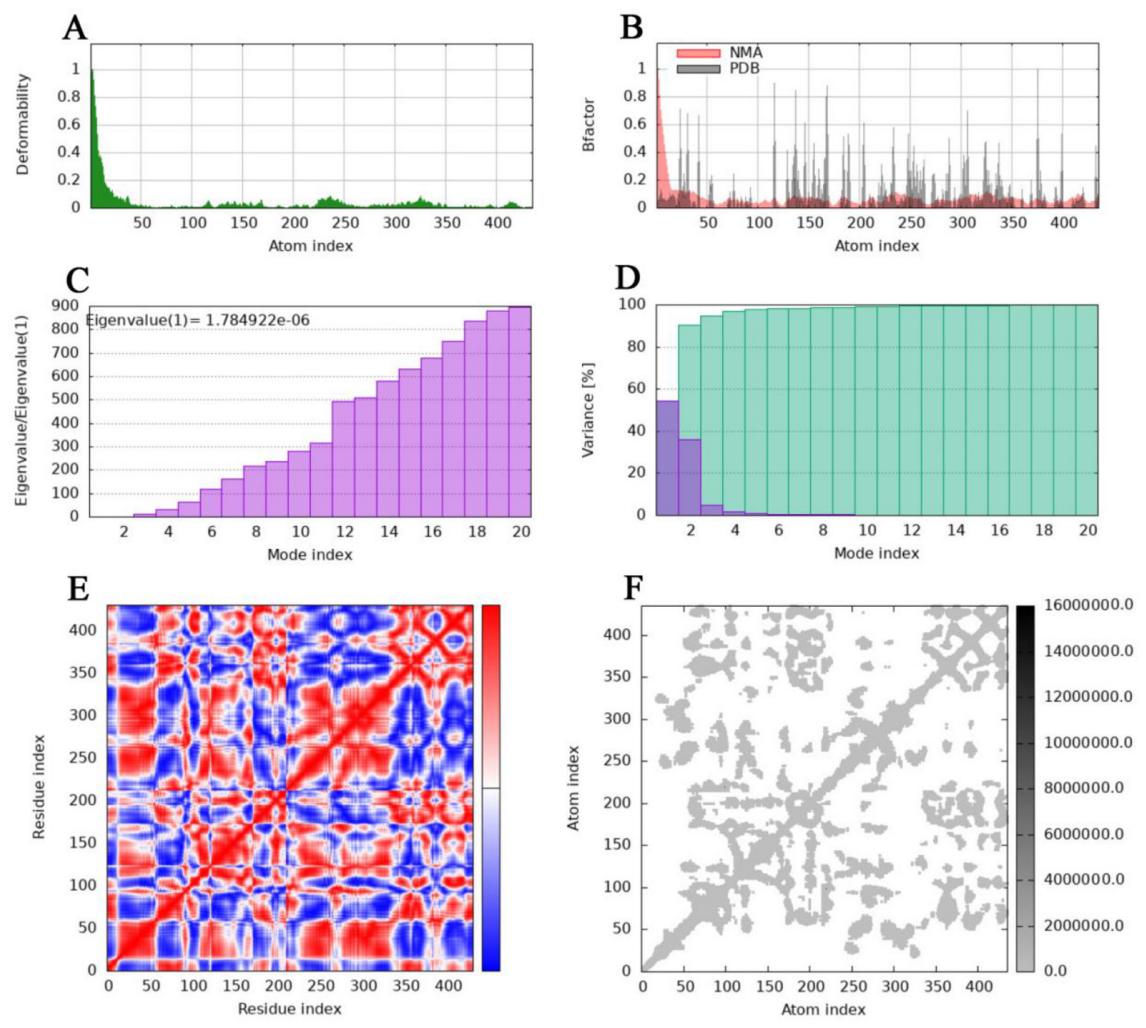
Molecular dynamics simulation of the NHase-SUL complex. **(A)** Main-chain deformability. **(B)** Experimental B-factor. **(C)** Eigenvalues. **(D)** Variance: green indicates cumulative variance, and **purple** indicates individual variance. **(E)** Covariance map: correlated motion is shown in **red**, and anticorrelated motion is shown in **blue**, respectively. **(F)** Elastic network.

The deformability and B-factors (Figures 9A, B, 10A, B) of the NHase-ACE and NHase-SUL complexes illustrate the peaks corresponding to the regions of the protein with deformability, where the highest peaks represent the regions of greatest deformability. The eigenvalue associated with each normal mode characterizes the modal stiffness, where lower eigenvalues indicate structural deformations requiring less energy, implying that modes are more easily excited. An eigenvalue of 1.78E−06 was calculated for both the NHase-ACE and NHase-SUL complexes (Figures 9C, 10C). The variance attributed to each normal mode is inversely proportional to the eigenvalue, where individual and cumulative variances are represented by red and green bars, respectively, further supporting the overall stability and dynamics characteristics of the NHase-ACE and NHase-SUL complex interaction (Figures 9D, 10D). The covariance matrix elucidates correlations between pairs of residues, with red, white, and blue colors indicating correlated, uncorrelated, and anti-correlated pairs, respectively (Figures 9E, 10E for the NHase-ACE and NHase-SUL complexes). The elastic network model specifies atomic pairs interconnected via springs. In the graph, each dot represents a spring linking a specific atom pair, with color intensity reflecting stiffness: darker grays denote higher stiffness, while lighter shades indicate softer springs (Figures 9F, 10F for the NHase-ACE and NHase-SUL complexes). The image shows that the color is light gray, indicating that the rigidity is not very strong.

## 4 Conclusion

Microbial remediation of co-contaminated organic pollutants is significantly more challenging than the remediation of a single organic pollutant. This is the first study on pure bacterial remediation of ACE and SUL co-contamination. Although the degradation activity of *Ensifer* sp. DA6 toward ACE and SUL requires further improvement, its unique capability to simultaneously degrade both organic pesticides holds significant potential for bioremediation applications. *Ensifer* sp. DA6 efficiently degraded ACE and SUL into IM-1-2 and X11719474 via the hydration pathway, with the cobalt-dependent NHase identified as the key enzyme responsible for this process. The enzymatic activity of the nitrile NHase was characterized, and a three-dimensional homology model of the enzyme was constructed. Immobilized *Ensifer* sp. DA6 cells effectively degrade ACE and SUL in Yellow River surface water, providing the foundation for microbial remediation of ACE-SUL co-pollution in surface water. Our findings reveal a bacterial mechanism for degrading ACE and SUL co-contamination. This study clarifies the role of microbes in degrading ACE and SUL environmental residues, identifying a bacterium with bioremediation potential for ACE-SUL co-contaminated sites.

## Data Availability

The datasets presented in this study can be found in online repositories. The names of the repository/repositories and accession number(s) can be found in the article/Supplementary material.
