# Supplementary material for: Bioremediation of acetamiprid and sulfoxaflor co-contamination by Ensifer sp. DA6 and characterization of a novel nitrile hydratase involved
Source: Front Microbiol. 2025 Nov 11;16:1705774. doi: 10.3389/fmicb.2025.1705774 (PMC12646544; doi:10.3389/fmicb.2025.1705774)
Supplement: Supplementary file 1 [file Data_Sheet_1.pdf]

Supplementary material

Bioremediation of insecticides acetamiprid and sulfoxaflor co-contamination by *Ensifer* sp. DA6 and characterization of the novel nitrile hydratase involved

Wenlong Yang[a, c*], Jia Kang[a, c], Yahui Shao[a, c], Yun Geng[a, c], Yingxin Zhang[a, c], Renlu Liu[b*], Gao Chen[a, c*]

[a]State Key Laboratory of Nutrient Use and Management, Shandong Academy of Agricultural Sciences, Jinan 250100, People's Republic of China.

[b]Key Laboratory of Jiangxi Province for Functional Biology and Pollution Control in Red Soil Regions, School of Life Sciences, Jinggangshan University, Ji'an 343009, People's Republic of China.

[c]Engineering Research Center of Jinan for Agricultural Microbial Resource Conservation and Biomanufacturing, Institute of Crop Germplasm Resources, Shandong Academy of Agricultural Sciences, Jinan 250100, People's Republic of China.

*Corresponding authors:

First corresponding authors:

Gao Chen, E-mail: gxchen001@hotmail.com

Second corresponding authors:

Renlu Liu, E-mail: liurenlu89@163.com

Third corresponding authors:

Wenlong Yang, E-mail: yangwenlong919@163.com

Figure S1. HPLC chromatogram of *Ensifer* sp. DA6-degraded SUL.

Figure S2. *Ensifer* sp. DA6 genomic circle graphs. (A) *Ensifer* sp. DA6 chromosome circle graph; (B) *Ensifer* sp. DA6 plasmid 1 circle diagram; (C) *Ensifer* sp. DA6 plasmid 2 circle graph.

Figure S3. Agarose gel electrophoresis of *Ensifer* sp. DA6 NHase gene amplification.

Figure S4. Full scan of the entire original agarose gel electrophoresis of *Ensifer* sp. DA6 NHase gene amplification.

Figure S5. Full scan of the entire original SDS-PAGE gel of NHase overexpressed in *E. coli* Rosetta (DE3) along with purified NHase.

Figure S6. Alignment of the NHase subunit alpha sequence with selected nitrile hydratase sequences. αCys-113, αCys-1116, αSer-117, and αCys-118 were predicted to be involved in cobalt ion binding and were marked in red.

Figure S7. Alignment of the NHase subunit beta sequence with selected nitrile hydratase sequences. βArg52 and βArg150 formed hydrogen bonds to stabilize the claw setting and were marked in red, βIle-48, βSer-51 and βTrp-72 were participated in the recognition of substrate by forming a hydrophobic pocket and were marked in green.

Figure S1.

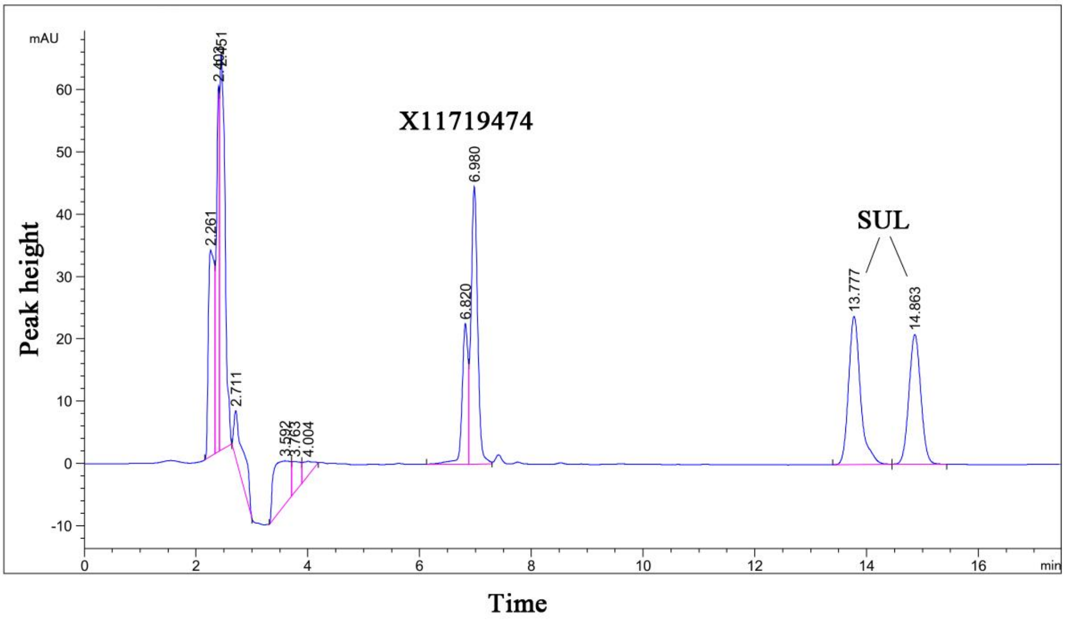

mAU
X11719474
SUL
Peak height
Time
min
2.261
2.711
6.820
6.980
13.777
14.863
3.592
3.763
4.004

Figure S2.

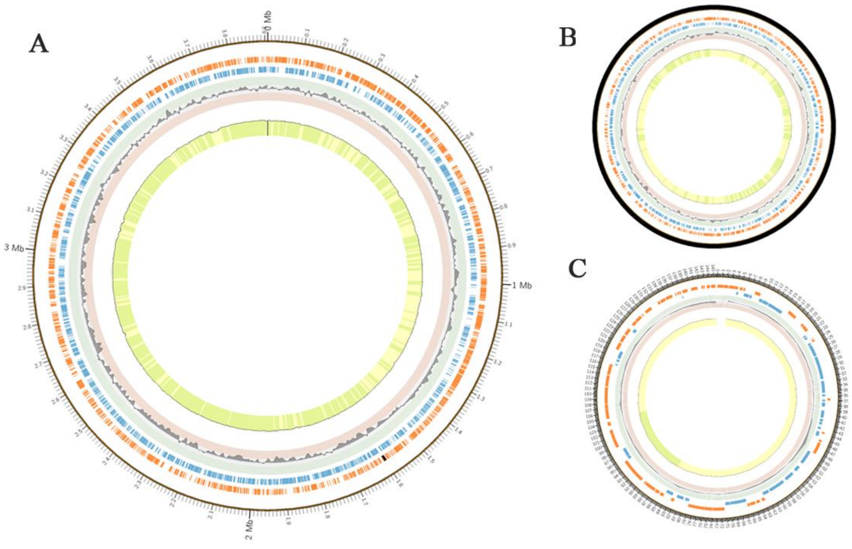

Figure S3.

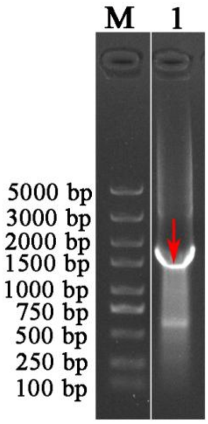
M
1
5000 bp
3000 bp
2000 bp
1500 bp
1000 bp
750 bp
500 bp
250 bp
100 bp

Figure S4.

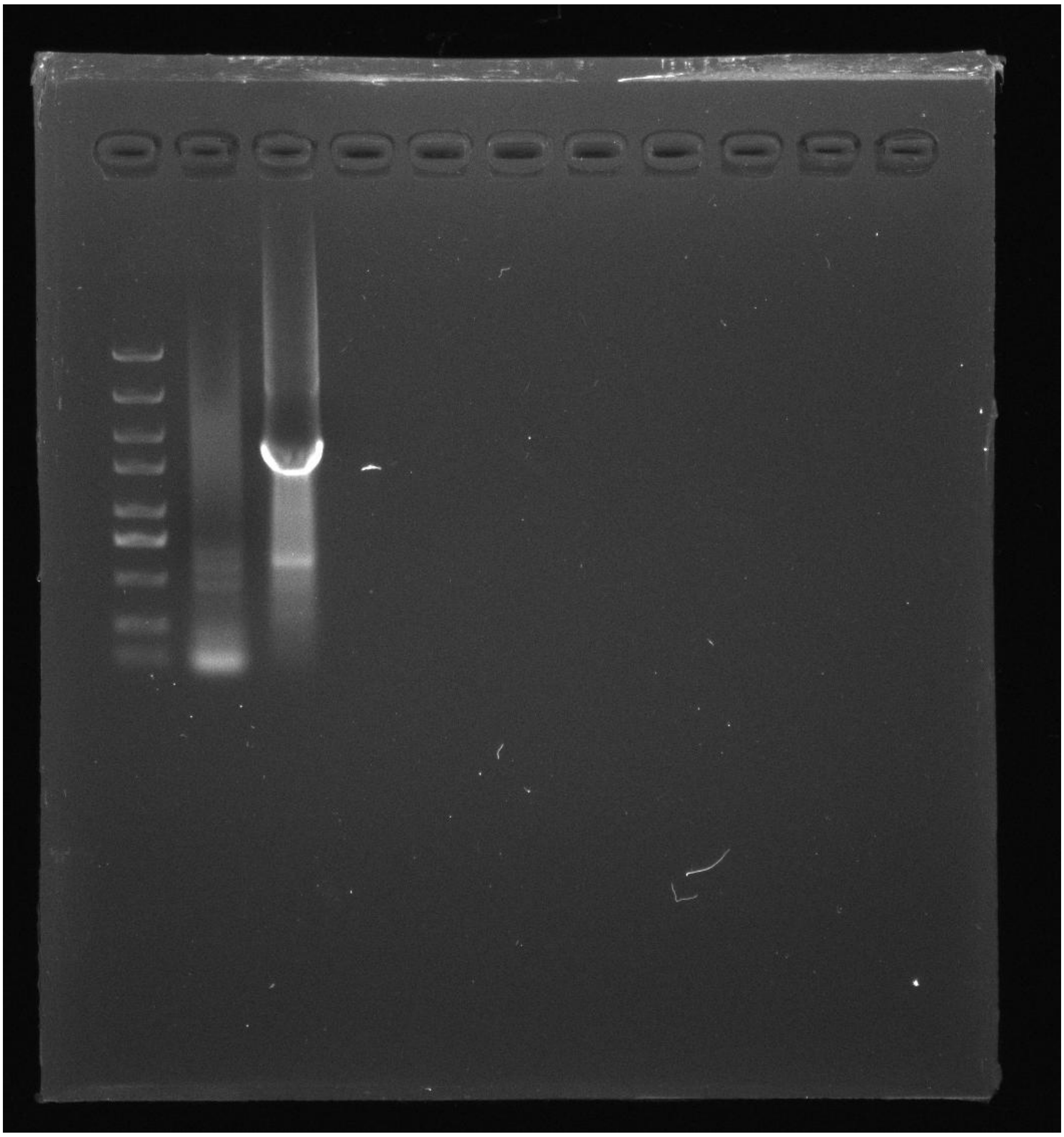

Figure S5.

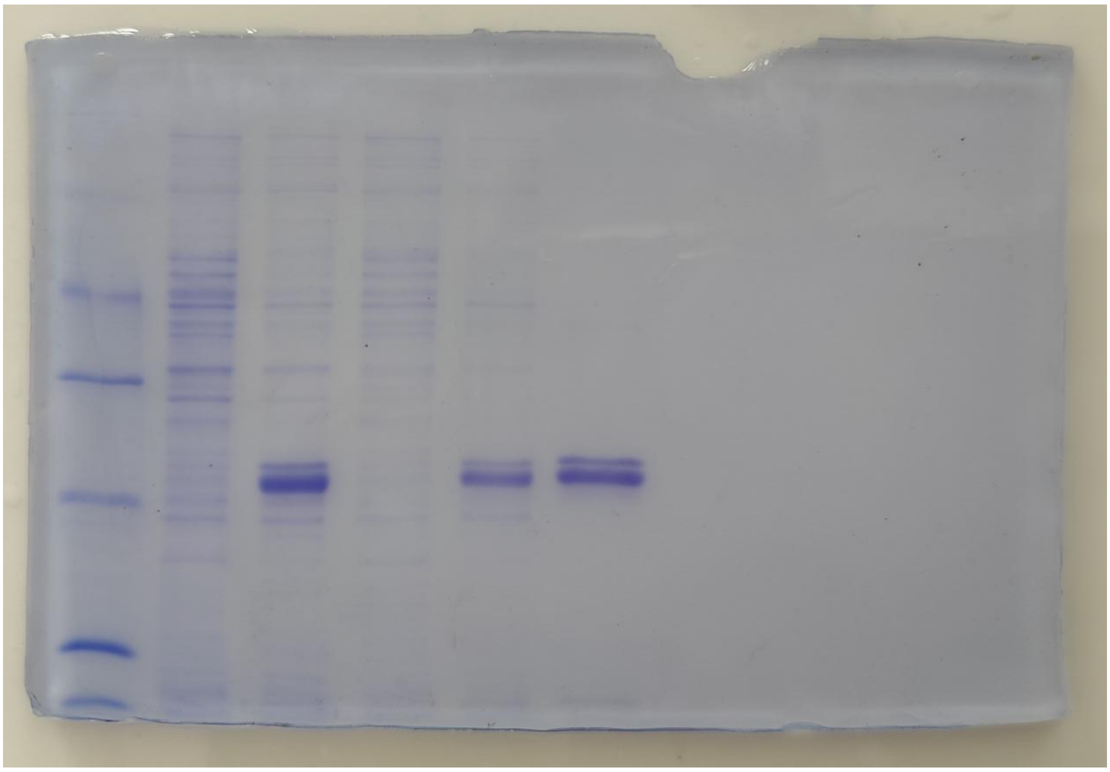

Figure S6.

***Ensifer* sp. DA6**	----------	MSEHHHGHGH	DRDHHDNHFT	-----DMEAR	VKALETVLTE
Pseudonocardia thermophila	----------	TENILRKSDE	EI--------	--QKEITARV	KALESMLIEQ
Pseudomonas putida	ASMTGGQQMG	RGSEFMGQSH	THDHHHDGYQ	A-PPEDIALR	VKALESLLIE
***Bacillus sp.* RAPc8**	--------MA	IEQKLMDDHH	EVDPRFPHHH	PRPQSFWEAR	AKALESLLIE
Rhodococcus erythropolis	----------	MSVTIDHTTE	NAAP------	--AQAPVSDR	AWALFRALDG
Pseudonocardia thermophila	----------	MTENILRKSD	EE--------	--IQKEITAR	VKALESMLIE
***Ensifer* sp. DA6**	KGLIDPAAID	AIVETYEMKI	GPRNGARVVA	KAWVDSDFAE	WLKRDATAAI
Pseudonocardia thermophila	GILTTSMIDR	MAEIYENEVG	PHLGAKVVVK	AWTDPEFKKR	LLADGTEACK
Pseudomonas putida	KGLVDPAAMD	LVVQTYEHKV	GPRNGAKVVA	KAWVDPAYKA	RLLADGTAGI
***Bacillus sp.* RAPc8**	KRLLSSDAIE	RVIKHYEHEL	GPMNGAKVVA	KAWTDPEFKQ	RLLEDPETVL
Rhodococcus erythropolis	KGLVPDGYVE	GWKKTFEEDF	SPRRGAELVA	RAWTDPEFRQ	LLLTDGTAAV
Pseudonocardia thermophila	QGILTTSMID	RMAEIYENEV	GPHLGAKVVV	KAWTDPEFKK	RLLADGTEAC
***Ensifer* sp. DA6**	ASLGFTGRQG	EHMRAVFNTA	DTHNLVVCTL	CSCYPWSVLG	LPPVWYKAPP
Pseudonocardia thermophila	ELGIGGLQGE	DMMWVENTDE	VHH-VVVCTL	CSCYPWPVLG	LPPNWFKEPQ
Pseudomonas putida	AELGFSGVQG	EDMVILENTP	AVHNVFVCTL	CSCYPWPTLG	LPPAWYKAAP
***Bacillus sp.* RAPc8**	RELGYFGLQG	EHIRVVENTD	TVHNVVVCTL	CSCYPWPLLG	LPPSWYKEPA
Rhodococcus erythropolis	AQYGYLGPQG	EYIVAVEDTP	TLKNVIVCSL	CSCTAWPILG	LPPTWYKSFE
Pseudonocardia thermophila	KELGIGGLQG	EDMMWVENTD	EVHHVVVCTL	CSCYPWPVLG	LPPNWFKEPQ
***Ensifer* sp. DA6**	YRSRAVIDPR	GVLSEFGVTL	PEATRIRVWD	STAELRYLVV	PERPAGTEGF
Pseudonocardia thermophila	YRSRVVREPR	QLLKEEFGFE	VPPSKEIKVW	DSSSEMRFVV	LPQRPAGTDG
Pseudomonas putida	YRSRMVSDPR	GVLAEFGLVI	PANKEIRVWD	TTAELRYMVL	PERPAGTEAY
***Bacillus sp.* RAPc8**	YRSRVVKEPR	KVLQEFGLDL	PDSVEIRVWD	SSSEVRFMVL	PQRPEGTEGM
Rhodococcus erythropolis	YRARVVREPR	KVLSEMGTEI	ASDIEIRVYD	TTAETRYMVL	PQRPAGTEGW
Pseudonocardia thermophila	YRSRVVREPR	QLLKEEFGFE	VPPSKEIKVW	DSSSEMRFVV	LPQRPAGTDG
***Ensifer* sp. DA6**	SEEELAGLVT	RDSMIGTGLA	LSAEAVR---	--↵	
Pseudonocardia thermophila	WSEEELATLV	TRESMIGVEP	AKAV------	--↵	
Pseudomonas putida	SEEQLAELVT	RDSMIGTGLP	TQPTPSH---	--↵	
***Bacillus sp.* RAPc8**	TEEELAQIVT	RDSMIGVAKV	QPPKVIQE--	--↵	
Rhodococcus erythropolis	SQEQLQEIVT	KDCLIGVAIP	QVPTV-----	--↵	
Pseudonocardia thermophila	WSEEELATLV	TRESMIGVEP	AKAVAHHHHH	H-↵	

Figure S7.

***Ensifer* sp. DA6**	MNGPHDLGGA	HGLGPVA--P	EKDE-PYFHA	EWEKRALGIT	LSCGAF--GA
Pseudonocardia thermophila	MNGVYDVGGT	DGLGPIN--R	PADE-PVFRA	EWEKVAFAMF	PATFRA--GF
Pseudomonas putida	MNGIHDTGGA	HGYGPVY--R	EPNE-PVFRY	DWEKTVMSLL	PALLAN--GN
***Bacillus sp*. RAPc8**	MNGIHDVGGM	DGFGKIMYVK	EEED-TYFKH	DWERLTFGLV	AGCMAQGLGM
Rhodococcus erythropolis	MDGVHDLAGV	QGFGKVPHTV	NADIGPTFHA	EWEHLPYSLM	FAGVAEL-GA
Pseudonocardia thermophila	MNGVYDVGGT	DGLGPIN--R	PADE-PVFRA	EWEKVAFAMF	PATFRA--GF

***Ensifer* sp. DA6**	WTIDESRHAR	ESLPPATYLS	ASYYEIWTRA	LETLLKRHGF	VSQAELDAGH
Pseudonocardia thermophila	MGLDEFRFGI	EQMNPAEYLE	SPYYWHWIRT	YIHHGVRTGK	IDLEELERRT
Pseudomonas putida	FNLDEFRHSI	ERMGPAHYLE	GTYYEHWLHV	FENLLVEKGV	LTATEVATG-
***Bacillus sp*. RAPc8**	KAFDEFRIGI	EKMRPVDYLT	SSYYGHWIAT	VAYNLLETGV	LDEKELEDRT
Rhodococcus erythropolis	FSVDEVRYVV	ERMEPRHYMM	TPFYERYVIG	VATLMVEKGI	LTQDELESL-
Pseudonocardia thermophila	MGLDEFRFGI	EQMNPAEYLE	SPYYWHWIRT	YIHHGVRTGK	IDLEELERRT

***Ensifer* sp. DA6**	RLEQGATPKR	VLKAD-----	--MVAGVLAK	GGPCDRPVET	APRFTVGDRV
Pseudonocardia thermophila	QYYRENPDAP	LPEHEQKPEL	IEFVNQAVYG	GLPASREVDR	PPKFKEGDVV
Pseudomonas putida	KAASGKTATP	VLTPA-----	--IVDGLLST	GASAAREEGA	RARFAVGDKV
***Bacillus sp*. RAPc8**	QAFMEKPDTK	IQRWE-NPKL	VKVVEKALLE	GLSPVREVSS	FPRFEVGERI
Rhodococcus erythropolis	----AGGPFP	LSRPS-----	-------ESE	GRPAPVETTT	---FEVGQRV
Pseudonocardia thermophila	QYYRENPDAP	LPEHEQKPEL	IEFVNQAVYG	GLPASREVDR	PPKFKEGDVV

***Ensifer* sp. DA6**	RTKNFNPETH	TRLPRYARAK	LGRVEA-VQG	SFVFPDDNAH	GRGEN-PQWV
Pseudonocardia thermophila	RFSTASPKGH	ARRARYVRGK	TGTVVK-HHG	AYIYPDTAGN	GLGEC-PEHL
Pseudomonas putida	RVLNKNPVGH	TRMPRYTRGK	VGTVVI-DHG	VFVTPDTAAH	GKGEH-PQHV
***Bacillus sp*. RAPc8**	KTRNIHPTGH	TRFPRYVRDK	YGVIEE-VYG	AHVFPDDAAH	RKGEN-PQYL
Rhodococcus erythropolis	RVRDEYVPGH	IRMPAYCRGR	VGTISHRTTE	KWPFPDAIGH	GRNDAGEEPT
Pseudonocardia thermophila	RFSTASPKGH	ARRARYVRGK	TGTVVK-HHG	AYIYPDTAGN	GLGEC-PEHL

***Ensifer* sp. DA6**	YTVVFDGPEI	WGEGVD-PTL	TVSIDAWESY	LEHV------	-
Pseudonocardia thermophila	YTVRFTAQEL	WGPEGD-PNS	SVYYDCWEPY	IELVDTC---	-
Pseudomonas putida	YTVSFTSVEL	WGQDASSPKD	TIRVDLWDDY	LEPA------	-
***Bacillus sp*. RAPc8**	YRVRFDAEEL	WGVK---QND	SVYIDLWEGY	LEPVSH----	-
Rhodococcus erythropolis	YHVKFAAEEL	FGSDTD--GG	SVVVDLFEGY	LEPAA-----	-
Pseudonocardia thermophila	YTVRFTAQEL	WGPEGD-PNS	SVYYDCWEPY	IELVDTKAAA	A
